# Urinogenital schistosomiasis knowledge, attitude, practices, and its clinical correlates among communities along water bodies in the Kwahu Afram Plains North District, Ghana

**DOI:** 10.1371/journal.pntd.0011513

**Published:** 2023-08-16

**Authors:** Samuel Essien-Baidoo, Mainprice Akuoko Essuman, Bernard Adarkwa-Yiadom, Dominic Adarkwa, Anita Akua Owusu, Seth Boakye Amponsah

**Affiliations:** 1 Department of Medical Laboratory Science, School of Allied Health Sciences, College of Health and Allied Sciences, University of Cape Coast, Cape Coast, Ghana; 2 Laboratory Department, Akomaa Memorial Adventist Hospital, Kortwia, Bekwai, Ghana; 3 Laboratory Department, Onwe Government Hospital, Ejisu-Onwe, Ghana; 4 Laboratory Department, Agona Nkwanta Health Centre, Agona Nkwanta, Ghana; Instituto Butantan, BRAZIL

## Abstract

**Background:**

Adequate knowledge and proper practices coupled with knowledge of the burden of disease are necessary for the eradication of *Schistosoma* infection. This study assessed knowledge, attitude, and practice (KAP) as well as health outcomes related to *Schistosoma haematobium* infection at Kwahu Afram Plains North District (KAPND).

**Methods:**

A cross-sectional survey using a structured questionnaire was carried out among 140 participants from four local communities in KAPND in August 2021. From these participants, 10ml of urine was collected for determination of the presence of *S*. *haematobium* and urine routine examination. In addition, 4ml of blood was collected and used for haematological examination. Descriptive statistics and logistic regression analysis using IBM SPSS were used to describe and represent the data collected.

**Results:**

The study reports a gap in knowledge about schistosomiasis in the study area with the majority indicating that they have not heard of schistosomiasis (60.7%), do not know the mode of transmission (49.3%), and do not know how the disease could be spread (51.5%). The overall prevalence of urinary schistosomiasis was 52.9%. This was associated with age, occupation, perceived mode of *Schistosoma* transmission, knowledge of *Schistosoma* prevention, awareness that schistosomiasis can be treated, frequency of visits to water bodies, and water usage patterns. In multivariate analysis, factors that remained significantly associated with *S*. *haematobium* infection were age 21–40 (OR  =  0.21, 95% CI: 0.06–0.76), 41–60 (OR  =  0.01, 95% CI: 0.01–0.52) and ≥ 60 (OR  =  0.02, 95% CI: 0.02–0.87), informal employment (OR  =  0.01, 95% CI: 0.01–0.69) and awareness of transmission by drinking water from river body (OR  =  0.03, 95% CI: 0.03–0.92). In *Schistosoma* infection, reduced haemoglobin, haematocrit, mean corpuscular volume, mean corpuscular haemoglobin, lymphocytes and eosinophils were observed. White blood cells, neutrophils, and monocytes were significantly elevated in infected states. Urine analysis revealed high pus cells and red blood cells counts among *Schistosoma*-positive participants.

**Conclusion:**

*Schistosoma* infection is endemic among inhabitants in KAPND, and is associated with a gap in knowledge, awareness, and practice possibly due to inadequate education in the area. Poor clinical outcomes associated with *Schistosoma* infection have been demonstrated in the area. A well-structured public education, nutritional intervention, and mass drug administration will be necessary to eradicate this menace.

## Author summary

Schistosomiasis is one of the leading neglected tropical diseases worldwide. Ghana is considered endemic to schistosomiasis and cited to be present throughout the whole country according to the 2015 World Schistosomiasis Risk Chart. Earlier studies have reported that individual and community perceptions of schistosomiasis are key factors that impact transmission rates and could help in planning and implementing control initiatives. In this study, we assessed community knowledge, attitudes, and practices related to schistosomiasis among inhabitants living along water bodies in the KAPND of Ghana. In addition, this study highlights the prevalence, risk factors, and impact of schistosomiasis in the area. We observed a gap in knowledge, attitude, and practice in the area. Consequently, a prevalence as high as 52.9% which was related to individual characteristics, knowledge, attitudes, and practices was observed. *Schistosoma* infection was associated with poor clinical outcomes in the area. Infection prevalence observed in the area indicates intense exposure and alarming transmission possibly due to poor knowledge, attitude, and practices. Control interventions targeted at controlling infection in the communities are warranted and should focus on educating inhabitants on *Schistosoma* prevention strategies and mass drug administration.

## 1. Introduction

Despite advancements in healthcare and globalization, *Schistosoma* infection remains a major global health issue confronting many people, especially children in developing countries. Schistosomiasis is an acute and chronic disease affecting over 230 million people worldwide with at least 218 million people estimated to require treatment [1,2]. Close to 85% of global cases are reported in Africa [2]. *S*. *haematobium*, which causes urogenital schistosomiasis, and *S*. *mansoni*, which causes intestinal schistosomiasis, are the two primary species infecting people in sub-Saharan Africa including Ghana [3]. *Schistosoma* infection has been ranked second as the most underestimated tropical disease related to poverty. It is responsible for over 534,000 deaths globally [4] with an estimated cost of 8 million disability-adjusted life years [5].

In Ghana, awareness and public concern for schistosomiasis go back to the 1960s during the construction of the Akosombo dam. The construction of the dam resulted in the sudden occurrence and infestation of *Bulinus truncatus* (an intermediate host of *S*. *haematobium*), resulting in urogenital schistosomiasis in several communities along the lake [6]. Additionally, the construction of the Kpong dam compounded this situation [7]. Ghana is still endemic to schistosomiasis and cited to be present throughout the whole country including urban areas according to the 2015 World Schistosomiasis Risk Chart [8]. *S*. *haematobium* infection in the country is variable, location-specific and depends on many factors including geographic location, gender, age, occupation, contact with water, distance to water resources and socioeconomic status [9].

Individual and community perceptions of schistosomiasis, as well as prevention and treatment, are key factors that impact transmission rates [10]. Early reports suggest that different control strategies should be designed keeping in mind the environmental situation and socioeconomic factors among people living in endemic communities [2,11]. However, information on knowledge, awareness and practice related to *Schistosoma* infection is scanty in some communities although very important in the formulation of public health campaigns intended to eradicate infection. For instance, in rural communities in the Kwahu Afram Plains North District (KAPND) of Ghana, where water contact activities are common, there is a paucity of information on community-based knowledge, attitude and practices (KAP) related to schistosomiasis. Additionally, there is limited information on the effect of *S*. *haematobium* infection on clinical outcomes in patients living in these endemic areas. This has the propensity to negatively impact the effectiveness and sustainability of control interventions aimed at eliminating schistosomiasis at the community level. There is the need therefore to further investigate and establish the burden of infection as this is necessary for public health intervention.

To this end, this study was undertaken with the following aims; (1) to estimate the prevalence and assess factors associated with *S*. *haematobium* infection at KAPND, (2) to assess the knowledge, attitudes and practices on schistosomiasis among inhabitants, and (3) to examine the effect of *Schistosoma* infection on haematological and urinary parameters of inhabitants, so that there can be a focused public health intervention for the control of *S*. *haematobium* in the district and other places.

## 2. Methods

### 2.1. Ethics statement

This study has been reported in accordance with the STROBES guidelines and the Declaration of Helsinki. Permission was obtained from traditional rulers for the communities as well. The purpose of the study was clearly explained to community leaders and participants for their permission and cooperation before their participation. Participants were invited to participate after verbal and written informed consent had been obtained. Participants were made to understand that their participation was voluntary and are free to redraw from the study at any point. Participants were given all details including: what the outcome of the study will be used for, their right to either give or withdraw their consent, data protection, and issues regarding confidentiality. All infected participants were counselled and referred to the district hospital for treatment.

### 2.2. Study design

This was a cross-sectional study conducted at Brumben, Amankwakrom, Agordeke Tonu and Donkorkrom which are communities along water bodies in the KAPND in the Eastern Region of Ghana. The selection of these sites was based on reports of cases of schistosomiasis-like symptoms, closeness to water bodies, increased water contacts activities (due to fishing, farming, swimming, and domestic use) and the lack of social amenities such as health, water and toilet facilities. Cross-sectional studies are the most effective technique to measure prevalence and are beneficial for discovering relationships that can subsequently be examined more thoroughly in a cohort study or randomized controlled trial [12].

### 2.3. Study area and population

**Fig 1:**
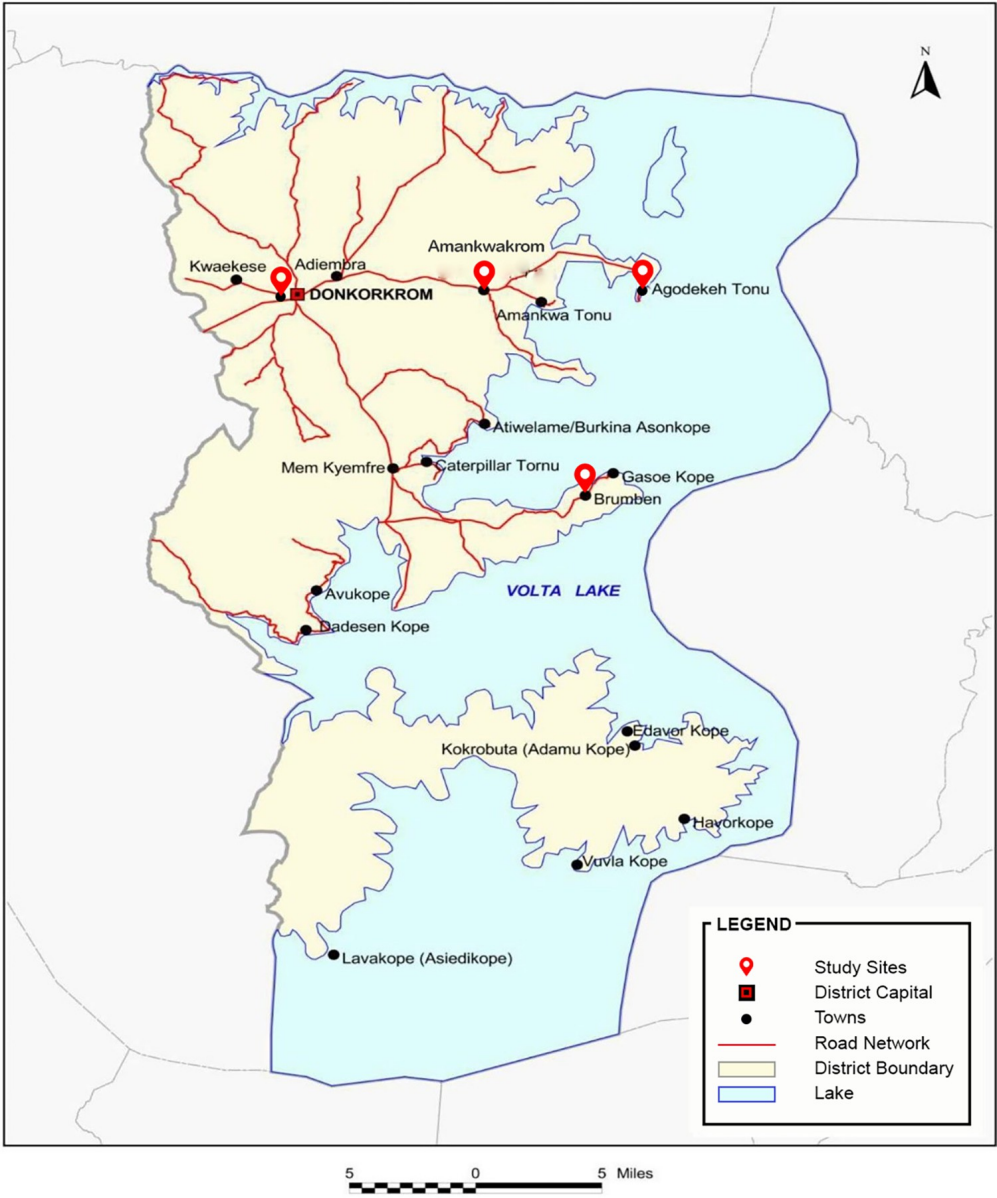
District map of Kwahu Afram Plains North district. Image credit: Ghana Statistical Service (https://www2.statsghana.gov.gh/docfiles/2010_District_Report/Eastern/KWAHU%20AFRAM%20PLAINS%20NORTH.pdf).

KAPND as described in the report of the 2010 population and housing census [13] has a total land area of approximately 2,341.3 km^2^ with Donkorkrom as its district capital. The area is surrounded by Kwahu Afram Plains South to the south, to the east with the Volta River, and to the west by two Districts in the Ashanti Region (Sekyere East and Asante-Akim Districts) (Fig 1). The population of KAPND is 102,423 representing 3.8% of the region’s total population. Out of this, 54,183 (52.9%) are males and 48,240 (47.1%) are females. The total population of the four communities (Brumben, Amankwakrom, Agordeke Tonu and Donkorkrom) selected for the study upon consultation with primary healthcare personnel was approximately 18,845. The land is generally undulating and rises about 60 meters to 120 meters above sea level. The only high ground is the Donkorkrom Plateau. The district is drained by the Afram River in the west, the Volta River in the east and the Obosom river in the north which flows continuously throughout the year and is used for farming, fishing, and household activities. As high as 72% of households in the district are engaged in agriculture and fishing activities.

### 2.4. Sample size determination and participants selection

Van Voorhis and Morgan’s general rule of thumb [14] which has been employed in similar studies [15] states that 30 individuals per group are needed to identify meaningful differences, which might result in 80% power. To improve the study’s reliability, 182 participants were included in this study. A disproportionate stratification based on the population size of each community was used to allocate a sample size of 40 each for Brumben, Amankwakrom, Agordeke Tonu and 62 for Donkorkrom.

We selected participants in a manner similar to earlier studies [16]. In each community, participants were recruited by randomly selecting households on a predetermined walking route based on a systematic sampling technique, and then knocking on doors. Specifically, we initially calculated a sample interval by dividing the total number of households in a community by the sample size of each community. Following that, we chose the first household at random using the fishbowl method of simple random sampling, and we chose successive houses at regular intervals. For each selected household, one resident (≥15 years) who agreed to voluntarily participate in the study and is a permanent resident in the community was enrolled. Where eligible adults willing to participate were more than one, a selection is made by balloting. If no adults fit our inclusion criteria in a household, the next household was selected. Individuals who declined to participate and confessed unwell, menstruating or having menstruated a few days before the time of the study were excluded.

### 2.5. Questionnaire survey

Questionnaires were administered to participants by three of the researchers, who helped to translate its content for those who could not understand over a twenty-day period (3^rd^ to 23^rd^ August 2021). Participants were administered a structured questionnaire to gather sociodemographic data including age, gender, and occupation. The questionnaire was designed by researchers based on existing studies on community KAP of schistosomiasis [17,18]. Aside from socioeconomic data taken, questions covered; (i) knowledge and attitudes related to schistosomiasis, its symptoms, and its prevention; (ii) attitudes and practices in relation to schistosomiasis prevention; (iii) health-seeking behaviours and (iv) whether the respondent had contracted schistosomiasis and or has received treatment in the past.

### 2.6. Sample collection and laboratory study

Participants provided freshly voided urine in clean, sterile, well labelled and leakproof containers between 10:00am to 2:00pm. Instructions on the procedure for collecting urine were given to the participants to ensure that contamination was avoided. A urine routine examination was performed using urine reagent dipsticks (URIT 10V, URIT Medical Electronic Co., Ltd. China). The manufacturer’s instructions were adhered to while taking note of blood, leucocyte and protein detection. Briefly, after immersing the test strip in the urine sample for 2 seconds, the strips were tapped to remove excess urine. Colour changes on reagent areas were then compared with the corresponding colour chart printed on the container label between 1–2 minutes. The results for blood were recorded as 0 (negative), 10 (trace), 25 (+), 80 (++) and ≥200 (+++) erythrocytes/μl urine. Protein was recorded as 0 (negative), 15 (trace), 30 (+), 100 (++) and 300 (+++) mg/dL while leucocyte was recorded as 0 (negative), 15 (trace), 70 (+), 125 (++) and 500 (+++) cell/μL urine.

Urine was centrifuged with a Biobase centrifuge (China) to obtain sediments for light microscopy. Sediments were analysed for *S*. *haematobium* by urine sedimentation technique. The number of *S*. *haematobium* eggs per 10 ml urine was determined for each participant [19]. The presence of pus cells and red blood cells were noted during the microscopy.

Finally, 4ml of venous blood was collected from participants into EDTA tubes for haematological analysis. Blood samples were analysed with a Sysmex automated haematology analyser (XN-350). The haematological profile (red blood cells, haemoglobin, haematocrit, red cell indices, white blood cells, platelets, and white blood cell differential count) was estimated after the control samples run. All samples were analysed within two hours of venepuncture. All laboratory analysis were carried out at the Donkorkrom Presbyterian Hospital Laboratory after samples have been transported in coolers containing ice packs.

### 2.7. Statistical analysis

Data was collected into Microsoft Excel and analysed using IBM SPSS version 26 (Armonk, NY, USA). The prevalence and intensity of infection were determined based on laboratory examination of urine samples. Frequencies and percentages of variables related to knowledge, attitude, and practices related to schistosomiasis were calculated to provide information on the KAPs of *S*. *haematobium* among the respondents. Continuous variables such as the age of participants, and the results of the haematological and urine examination were presented using mean and standard deviation. Differences among participants who were positive and negative for *S*. *haematobium* were assessed by the Chi-square or Fisher exact test for categorical variables and the students t-test for numerical variables. Associations were explored between a set of indicators relating to levels of KAP, socio-demographic characteristics and *Schistosoma* infection using univariate logistic regression. To obtain adjusted odds ratios, all independent variables with a P-value ≤ 0.25 were subjected to multivariate logistic regression analysis. P-values less than 0.05 was considered statistically significant in all analysis.

## 3. Results

### 3.1. Participant characteristics and prevalence of schistosomiasis

In this cross-sectional study, 182 inhabitants were enrolled. However, 22 participants did not give any or inadequate urine for parasitological and urine examination, and 20 participants had no or incomplete questionnaire data. As a result, the final analysis included 140 individuals (made up of 33 males and 107 females) who completed the questionnaire and provided enough urine samples. The average age of participants was 32.82±15.11 with the youngest being 15 years and the oldest being 79 years. The overall prevalence of *S*. *haematobium* among participants was 52.9%. Infection intensities was light (mean: 5.71±7.0, between 4–32 eggs/10ml urine) for all participants. *Schistosoma*-positive participants were significantly younger than *Schistosoma* negative participants (29.68 ± 14.08 vs 36.35 ± 15.55, p = 0.009). Many of the study participants were between ages 21 to 40 (57.9%), and worked in the informal sector (62.1%), with statistically significant differences between ages and occupation of *Schistosoma* positive and negative participants (Table 1).

**Table 1:** Socio-demographic characteristics of study participants.

Variable	Total (n = 140)	*S*. *haematobium* positive individuals (n = 74)	*S*. *haematobium* negative individuals (n = 66)	P-value
Average age (years)	32.82 ± 15.11	29.68 ± 14.08	36.35 ± 15.55	**0.009**
Age groups (years)				**0.012**
≤ 20	29 (20.7)	22 (29.7)	7 (10.6)	
21–40	81 (57.9)	42 (56.8)	39 (59.1)	
41–60	17 (12.1)	5 (6.8)	12 (18.2)	
> 60	13 (9.3)	5 (6.8)	8 (12.1)	
Gender				0.860
Male	33 (23.6)	17 (23.0)	16 (24.2)	
Female	107 (76.4)	57 (77.0)	50 (75.8)	
Occupation				**0.018**
Formal	41 (29.3)	21 (28.4)	20 (30.3)	
Informal	87 (62.1)	42 (56.8)	45 (68.2)	
Unemployed	12 (8.6)	11 (14.9)	1 (1.5)	

Values are presented as frequency (percentage) for categorical variables and mean ± standard deviation for continuous variables.

### 3.2. Knowledge of *Schistosoma* infection

Participants demonstrated inadequate knowledge of schistosomiasis as the majority indicated that they have not heard of schistosomiasis (60.7%), do not know the mode of transmission (49.3%) and do not know how the disease could be spread (52.9%). Many participants mentioned that blood in urine (64.4%) is the main symptom of infection (Table 2).

**Table 2:** Responses of participants regarding their knowledge of schistosomiasis.

Question	Response rate (%)		
	Male	Female	Total
Heard of schistosomiasis			
Yes	12 (36.4)	43 (40.2)	55 (39.3)
No	21 (63.6)	64 (59.8)	85 (60.7)
Perceived mode of transmission			
Contact with water	3 (9.1)	19 (17.8)	22 (15.7)
Drinking water from river	14 (42.4)	32 (29.9)	46 (32.9)
Inherited from parents	1 (3.0)	2 (1.9)	3 (2.1)
Don’t know	15 (45.5)	54 (50.5)	69 (49.3)
Awareness of symptoms of schistosomiasis			
Blood in stool	0 (0.0)	4 (6.0)	4 (4.6)
Blood in urine	15 (75.0)	41 (61.2)	56 (64.4)
Rashes	1 (5.0)	5 (7.5)	6 (6.9)
Swollen abdomen	3 (15.0)	11 (16.4)	14 (16.1)
Itchy skin	0 (0.0)	1 (1.5)	1 (1.1)
Others	1 (5.0)	5 (7.5)	6 (6.9)
Awareness of schistosomiasis transmission			
Eating with others	0 (0.0)	2 (1.9)	2 (1.4)
Washing in river	5 (15.2)	8 (7.5)	13 (9.3)
Defecating in river	3 (9.1)	15 (14.0)	18 (12.9)
Bathing in river	7 (21.2)	26 (24.3)	33 (23.6)
Don’t know	18 (54.5)	56 (52.3)	74 (52.9)
Awareness of schistosomiasis prevention			
Avoid water contact activities	5 (15.2)	11 (10.3)	16 (11.4)
Avoid defecating in river	3 (9.1)	8 (7.5)	11 (7.9)
Avoid bathing in river	2 (6.1)	22 (20.6)	24 (17.1)
Weeding around water bodies	0 (0.0)	3 (2.8)	3 (2.1)
Don’t know	23 (69.7)	63 (58.9)	86 (61.4)

### 3.3. Attitude and health-seeking behaviours towards *Schistosoma* infection

Assessment of attitudes toward schistosomiasis, including participants health-seeking behaviors, showed that most participants (65.7%) considered schistosomiasis as a serious disease, and believe that it is possible to prevent (88.6%). Less than half of participants (48.6%) believe that the disease can be treated. The majority (63.6%, n = 89) indicated that they have not been infected by *Schistosoma* before. However, 23 (45.1%) of those who have been infected before indicated that they visited the hospital for treatment. Two-third of those who did nothing when they had infection stated that they did not seek for medical attention because they lacked funds (Table 3).

**Table 3:** Responses of participants regarding their attitude and health-seeking behaviours.

Question	Response rate (%)		
	Male	Female	Total
Believe that *Schistosoma* is a serious disease			
Yes	23 (69.7)	69 (64.5)	92 (65.7)
No	10 (30.3)	38 (35.5)	48 (34.3)
Believe that *Schistosoma* can be prevented			
Yes	26 (78.8)	98 (91.6)	124 (88.6)
No	7 (21.2)	9 (8.4)	16 (11.4)
Believe that *Schistosoma* can be treated			
Yes	12 (34.6)	56 (52.3)	68 (48.6)
No	7 (21.2)	17 (15.9)	24 (17.1)
Don’t know	14 (42.4)	34 (31.8)	48 (34.3)
Previously contracted schistosomiasis			
Yes	12 (36.4)	39 (36.4)	51 (36.4)
No	21 (63.6)	68 (63.4)	89 (63.6)
Action taken to treat schistosomiasis			
Visited the hospital	6 (50.0)	17 (43.6)	23 (45.1)
Visited a native doctor	4 (33.3)	9 (23.1)	13 (25.5)
Nothing	2 (16.7)	13 (33.3)	15 (29.4)
Reason for doing nothing to treat infection			
It is normal	1 (50.0)	4 (36.4)	5 (33.3)
Lack of funds	1 (50.0)	9 (63.6)	10 (66.7)

### 3.4. Behavioural practices related to *Schistosoma* infection

Table 4 summarizes the responses of participants to questions which assessed their behavioural practices. More than half (55.7%) of the study participants do not have clear water contact sites. Of these, majority (61.5%) believe it is a communal work. The majority (67.9%) visits rivers very often. More than half of the participants have seen blood in their urine before. The majority (45.7%) of those who have seen blood in their urine before indicated that it may be because of intake of water from the stream. Only a few (18/140) have received *Schistosoma* treatment before of which most (9/18) received it between 3–9 months ago.

**Table 4:** Responses of participants regarding their behavioural practices related to *Schistosoma* infection.

Question	Response rate		
	Male	Female	Total
Clearing of bushes around water contact sites			
Yes	12 (36.4)	50 (46.7)	62 (44.3)
No	21 (63.6)	57 (53.3)	78 (55.7)
Reason for not clearing bushes around water contact site			
It is a communal work	14 (66.7)	34 (59.6)	48 (61.5)
People have been paid to clear it	5 (23.8)	17 (29.8)	22 (28.2)
It is not important	1 (4.8)	3 (5.3)	4 (5.1)
Other reasons	1 (4.8)	3 (5.3)	4 (5.1)
Activities carried out in water bodies			
Fishing	10 (30.3)	30 (28.0)	40 (28.6)
Swimming	2 (6.1)	19 (17.8)	21 (15.0)
Bathing	14 (42.7)	45 (42.1)	59 (42.1)
Washing	7 (21.2)	13 (12.1)	20 (14.3)
Frequency of visit to water bodies			
Very often	21 (63.6)	74 (69.2)	95 (67.9)
Once a week	9 (27.3)	28 (26.2)	37 (26.4)
Once a month	3 (9.1)	5 (4.7)	8 (5.7)
Water usage			
Drinking	7 (21.2)	26 (24.3)	33 (23.6)
Cooking	7 (21.2)	24 (22.4)	31 (22.1)
Washing	6 (18.2)	25 (23.4)	31 (22.1)
Bathing	13 (39.4)	32 (29.9)	45 (32.1)
Seen blood in urine before			
Yes	22 (66.7)	59 (55.1)	81 (57.9)
No	11 (33.3)	48 (44.9)	59 (42.1)
Awareness of cause of the blood in urine			
Witchcraft	3 (13.6)	13 (22.0)	16 (19.8)
Curse	4 (18.2)	14 (23.7)	18 (22.2)
Adulthood	3 (13.6)	7 (11.9)	10 (12.3)
Drinking stream water	12 (54.5)	25 (42.4)	37 (45.7)
Has previously participated in mass treatment			
Yes	5 (15.2)	13 (12.1)	18 (12.9)
No	28 (84.8)	94 (87.9)	122 (87.1)
Period for last treatment received			
<3months	3 (60.0)	5 (38.5)	8 (44.4)
3–9 months	2 (40.0)	7 (53.8)	9 (50.0)
>9 months	0 (0.0)	1 (7.7)	1 (5.6)

### 3.5. Association of demographics and KAP with *S*. *haematobium* infection

Table 5 summarizes the univariate and multivariate analysis of the factors associated with *S*. *haematobium* infection at KAPND. The transmission of urogenital schistosomiasis was associated with age, occupation, perceived mode of *Schistosoma* transmission, knowledge of *Schistosoma* prevention and awareness of schistosomiasis treatment. Other significant factors included frequency of visit to water bodies, as well as water usage patterns. After adjusting for the confounders, the odds of *S*. *haematobium* infection was lower among respondents aged 21–40 (OR  =  0.21, 95% CI: 0.06–0.76), 41–60 (OR  =  0.01, 95% CI: 0.01–0.52) and ≥ 60 (OR  =  0.02, 95% CI: 0.02–0.87) than younger groups (≤ 20). Participants who were informally employed were less likely to be infected (OR  =  0.01, 95% CI: 0.01–0.69) as compared to those unemployed. In addition, participants who were aware that transmission could be acquired by drinking water from river body were (OR  =  0.03, 95% CI: 0.03–0.92) were less likely to be infected than those who were unaware of the mood of transmission.

**Table 5:** Logistic regression analysis of factors associated with *S*. *haematobium* among study participants.

Variable	Univariate analysis		Multivariate analysis	
	cOR	p-value	aOR	p-value
Age				
≤ 20	1 (reference)		1 (reference)	
21–40	0.29 (0.12–0.71)	0.007	0.21 (0.06–0.76)	0.017
41–60	0.09 (0.02–0.35)	<0.001	0.07 (0.01–0.52)	0.010
> 60	0.18 (0.05–0.70)	0.013	0.12 (0.02–0.87)	0.036
Gender				
Male	1 (reference)			
Female	1.07 (0.49–2.34)	0.860		
Occupation				
Unemployed	1 (reference)		1 (reference)	
Formal	0.10 (0.01–0.81)	0.031	0.21 (0.02–2.63)	0.226
Informal	0.09 (0.01–0.69)	0.021	0.06 (0.01–0.69)	0.023
Heard of schistosomiasis				
No	1 (reference)			
Yes	0.69 (0.35–1.37)	0.288		
Perceived mode of transmission				
Don’t know	1 (reference)		1 (reference)	
Contact with water	0.26 (0.10–0.72)	0.009	0.34 (0.05–2.59)	0.297
Drinking water from river	0.17 (0.07–0.38)	<0.001	0.16 (0.03–0.92)	0.040
Inherited from parents	0.19 (0.02–2.22)	0.185	0.60 (0.02–14.60)	0.750
Awareness of schistosomiasis transmission				
Don’t know	1 (reference)		1 (reference)	
Eating with others	0.6 (0.35–1.1)	0.669	1.78 (0.24–4.3)	0.918
Washing in river	0.11 (0.02–0.54)	0.006	0.42 (0.04–4.52)	0.478
Defecating in river	0.49 (0.17–1.38)	0.176	1.40 (0.21–9.39)	0.727
Bathing in river	0.73 (0.32–1.68)	0.459	1.89 (0.36–9.90)	0.453
Believe that *Schistosoma* can be prevented				
No	1 (reference)		1 (reference)	
Yes	2.02 (0.69–5.91)	0.197	1.62 (0.33–7.91)	0.549
Awareness of schistosomiasis prevention				
Don’t know	1 (reference)		1 (reference)	
Avoid contact with water	0.28 (0.09–0.89)	0.030	0.36 (0.07–1.82)	0.214
Avoid defecating in river	1.09 (0.30–4.01)	0.897	0.37 (0.06–2.32)	0.286
Avoid bathing in river	0.37 (0.15–0.95)	0.039	0.37 (0.10–1.45)	0.154
Weeding around water bodies	0.66 (0.19–1.7)	0.399	0.38 (0.38–2.71)	0.256
Believe that *Schistosoma* can be treated				
Don’t know	1 (reference)		1 (reference)	
Yes	0.35 (0.16–0.78)	0.010	1.11 (0.25–4.97)	0.897
No	0.12 (0.04–0.38)	<0.001	0.35 (0.05–2.36)	0.282
Previously contracted schistosomiasis				
No	1 (reference)			
Yes	0.69 (0.35–1.38)	0.299		
Clearing of bushes around water contact sites				
No	1 (reference)			
Yes	1.30 (0.66–2.53)	0.448		
Activities carried out in water bodies				
Fishing	1 (reference)			
Swimming	0.56 (0.19–1.64)	0.287		
Bathing	1.32 (0.59–2.96)	0.502		
Washing	0.91 (0.31–2.65)	0.855		
Frequency of visit to water bodies				
Very often	1 (reference)		1 (reference)	
Once a week	0.28 (0.13–0.63)	0.002	0.37 (0.10–1.39)	0.141
Once a month	0.19 (0.04–1.02)	0.052	1.14 (0.09)	0.921
Water usage				
Drinking	1 (reference)		1 (reference)	
Cooking	2.79 (1.00–7.79)	0.050	1.44 (0.27–7.69)	0.673
Washing	3.19 (1.14–8.92)	0.027	2.15 (0.47–9.90)	0.326
Bathing	4.17 (1.59–10.90)	0.004	1.82 (0.41–8.13)	0.436
Seen blood in urine				
No	1 (reference)		1 (reference)	
Yes	0.50 (0.25–0.99)	0.047	0.49 (0.15–1.54)	0.219
Has previously participated in mass treatment				
No	1 (reference)		1 (reference)	
Yes	0.40 (0.14–1.13)	0.083	1.24 (0.22–7.01)	0.808

*Abbreviations*: cOR, crude odds ratio; aOR, adjusted odds ratio; CI, confidence interval

### 3.6. Effect of *Schistosoma* infection on haematological parameters

Table 6 summarises summarizes haematological parameters in *Schistosoma* positive and negative participants. *Schistosoma* positive participants had significantly reduced levels of haemoglobin (10.47±2.29, p = 0.020), haematocrit (32.15±6.34, p = 0.002), mean corpuscular volume (78.61±9.97, p = 0.007), mean corpuscular haemoglobin (25.51±3.65, p = 0.005), lymphocytes (25.47±13.54, p = 0.017) and eosinophils (2.31±3.26, p = 0.009) than participants without schistosomiasis. In addition, white blood cells (9.64±4.18 vs 7.55±3.19, p = 0.001), neutrophils (6.48±3.74 vs 4.43±2.82, p<0.001) and monocytes (0.73±0.40 vs 0.59±0.28, p = 0.026) were significantly elevated in *Schistosoma* infection states.

**Table 6:** Effects of *Schistosoma* infection on haematological parameters of participants.

Parameter	Total	*S*. *haematobium* positive individuals	*S*. *haematobium* negative individuals	p-value
Haemoglobin (g/dL)	11.03 ± 2.30	10.47 ± 2.29	11.67 ± 2.16	**0.020**
Red blood cells (x10^12^/L)	4.22 ± 0.83	4.12 ± 0.84	4.34 ± 0.81	0.120
Haematocrit (%)	33.78 ± 6.51	32.15 ± 6.34	35.6 ±6.25	**0.002**
Mean cell volume (fL)	80.51 ± 8.90	78.61 ± 9.97	82.64 ± 7.0	**0.007**
Mean cell haemoglobin (pg)	26.25 ± 3.34	25.51 ± 3.65	27.09 ± 2.74	**0.005**
Mean cell haemoglobin concentration (g/dL)	32.55 ± 1.70	32.36 ± 1.87	32.76 ± 1.47	0.168
Red cell distribution width -SD (%)	41.26 ± 6.25	41.22 ± 6.73	41.30 ± 5.73	0.945
Red cell distribution width—CV (%)	14.16 ± 2.48	14.60 ± 2.89	13.68 ± 1.81	**0.028**
Platelet count (x10^12^/L)	252.86 ± 94.40	262.55 ± 107.63	242.00 ± 76.30	0.200
Platelet distribution width (%)	10.95 ±2.24	10.89 ± 2.14	11.02 ± 2.36	0.740
Mean platelet volume (fL)	9.90 ± 1.03	9.88 ± 1.00	9.92 ± 1.08	0.820
Plateletcrit (%)	0.25 ± 0.08	0.26 ± 0.09	0.24 ± 0.07	0.180
Total white blood cells (x10^9^/L)	8.66 ± 3.88	9.64 ± 4.18	7.55 ± 3.19	**0.001**
Neutrophils (%)	60.31 ± 16.17	64.16 ± 15.88	56.00 ± 15.50	**0.003**
Lymphocytes (%)	27.95 ± 13.05	25.47 ± 13.54	30.73 ± 11.98	**0.017**
Monocytes (%)	7.91 ± 2.64	7.64 ± 2.50	8.20 ± 2.78	0.208
Eosinophils (%)	3.20 ± 4.32	2.31 ± 3.26	4.20 ± 5.10	**0.009**
Basophils (%)	0.43 ± 0.46	0.44 ± 0.59	0.42 ± 0.25	0.758

Values are presented as mean ± standard deviation.

### 3.7. Effect of schistosomiasis on urine parameters

Urine analysis results revealed a statistically significant differences among *Schistosoma* positive and negative individuals. Pus cells (20.93 ± 19.42 vs 11.02 ± 23.36, p = 0.007) and red blood cells (34.01 ± 28.21 vs 4.27 ± 15.92, p<0.001) counts were significantly elevated in *Schistosoma* positive individuals. In addition, urine protein, blood and leucocytes were predominantly detected in urine samples of *S*. *haematobium* positive individuals (Table 7).

**Table 7:** Urine analysis results of participants with and without schistosomiasis.

Parameter	Total	*S*. *haematobium* positive individuals	*S*. *haematobium* negative individuals	p-value
Pus cells	16.26 ± 21.86	20.93 ± 19.42	11.02 ± 23.36	**0.007**
RBC/HPF	19.99 ± 27.54	34.01 ± 28.21	4.27 ± 15.92	**<0.001**
Urine Protein				**<0.001**
Negative	57 (40.7)	16 (21.6)	41 (62.1)	
Trace	15 (10.7)	3 (4.1)	12 (18.2)	
Positive (+)	24 (17.1)	13 (17.6)	11 (16.7)	
Positive (++)	38 (27.1)	37 (50.0)	1 (1.5)	
Positive (+++)	6 (4.3)	5 (6.8)	1 (1.5)	
Blood in Urine				**<0.001**
Negative	52 (37.1)	0 (0.0)	52 (78.8)	
Trace	3 (2.1)	0 (0)	3 (4.5)	
Positive (+)	8 (5.7)	7 (9.5)	1 (1.5)	
Positive (++)	43 (30.7)	41 (55.4)	2 (3.0)	
Positive (+++)	34 (24.3)	26 (35.1)	8 (12.1)	
Leukocytes				**<0.001**
Negative	35 (25.0)	3 (4.1)	32 (48.5)	
Trace	17 (12.1)	2 (2.7)	15 (22.7)	
Positive (+)	29 (20.7)	24 (32.4)	5 (7.6)	
Positive (++)	34 (24.3)	29 (39.2)	5 (7.6)	
Positive (+++)	25 (17.9)	16 (21.6)	9 (13.6)	

*Abbreviations*: RBC, red blood cells; HPF, high power field.

## 4. Discussion

Urogenital schistosomiasis is one of the most prevalent chronic infections among under-developed nations in the world, known as neglected tropical diseases, with 93% of the world’s 207 million schistosomiasis infections being recorded in sub-Saharan Africa, including 15 million cases in Ghana [20]. Despite attempts to eliminate schistosomiasis through interventions such as mass drug administration initiatives, prevalence is still a problem in some communities in Africa. This study assessed knowledge, awareness, practice as well as clinical outcomes of urogenital schistosomiasis among people living at Brumben, Amankwakrom, Agordeke Tonu and Donkorkrom communities in the KAPND in the Eastern Region of Ghana.

The study reports a prevalence as high as 52.9% observed among study participants. This number does not significantly differ from an earlier report among an adult population at the Volta Basin of 46.5% [21] but was much higher than 15.5%, 34.4% and 20.7% earlier reported at villages around Accra [22], Akotokyir in Cape Coast Municipal [23] and Kumasi [24] respectively. However, the reported prevalence was lower than 76% and 77.3% earlier reported at Weija community and Awutu-Efutu Senya District respectively [25,26]. Schistosomiasis infection is focal in distribution; hence variations in prevalence rates are expected. The high prevalence of *S*. *haematobium* infection observed in the area is worrying and could be attributed to the heavy dependence on the Afram River, Volta River and the Obosom River for fishing, water supply, transportation, and recreation. This has been spearheaded by poor access to proper water and sanitation facilities and reduced health coverage in the district [27]. Additionally, poor awareness, knowledge and behavioural practices as observed in this study could be responsible for the observed prevalence among inhabitants. Differences in prevalence may be attributed to the localities studied, availability of water bodies, water contact activities, age of study participants, occupation of inhabitants as well as period of the study. In the present study, infection was associated with age, occupation, frequency of visit to water bodies and water usage patterns similar to earlier reports [28,29]. The association of schistosomiasis with knowledge of *Schistosoma* transmission, prevention and treatment corroborates findings from earlier studies [28,29] and highlights the need to intensify public education.

The study further highlights a gap in knowledge about schistosomiasis in the study area as participants demonstrated inadequate knowledge of schistosomiasis with majority indicating that they have not heard of schistosomiasis, do not know the mode of transmission, and do not know how the disease could be spread. This observation was not surprising as prevalence was equally on the rise at the area. Comprehensive information, good attitudes, and practices have been shown to have a favourable impact on parasitic infection prevention and control [30,31]. Unlike our report in this study, appreciable knowledge and awareness were reported among people living around the Volta Basin of Ghana [21]. *Schistosoma* infection has long been reported to be prevalent in the Volta Basin [6] and may explain the high awareness in the area possibly due to continued educational campaigns in the area. Increased awareness through public education is needed for complete eradication of *Schistosoma* infection [2,31,32] and when properly conducted, would be necessary for *Schistosoma* control among the study population.

Belief in the seriousness and preventability of the disease was revealed to be a key attitude in this study. The findings from the study revealed that participants generally believed that schistosomiasis is a serious disease and can be prevented with a moderate number of them believing that it can be treated (Table 3). High recognition of the seriousness of the disease translated into proper health-seeking behaviours unlike an earlier report from Nigeria [33]. We observed that the majority of participants indicated that they had not been infected before which was not surprising as study participants demonstrated inadequate knowledge of schistosomiasis. However, a larger portion of those who indicated being infected before visited hospital/clinic for treatment which is plausible. Sadly, over two third of those who did nothing when they had been infection stated that they didn’t seek for medical attention because they lacked funds. Similar to our findings, an overwhelming 74.4% of respondents were reported to indicate that they did not seek healthcare because cost of treatment is expensive [21]. This observation is particularly worrying in the fight against mortality and morbidity due to neglected tropical diseases. It is important that treatment for *Schistosoma* infection be included in the National Health Insurance Scheme (NHIS) to take off the burden of these inhabitants who are mostly in the informal sector and may not be able to foot the cost of medical consultation, diagnosis, and treatment.

Although several studies have been conducted on the prevalence and dynamics of schistosomiasis in Ghana and other places, data on the effect of infection on participant’s haematological profile and urine analysis results is rare. To further understand the clinical impact of schistosomiasis, the impact on clinical outcomes was assessed. The study reports significantly lower levels of HGB, HCT, MCV, MCH, LYM and EOS and higher levels of white blood cells, neutrophils and monocytes in participants infected with *Schistosoma*. This observation corroborates findings from an earlier study conduct at Yeji, in the Atebubu District of Brong Ahafo Region, Ghana [34]. Low haemoglobin has been reported among individuals with schistosomiasis and have been suggestive of anaemia among people in endemic areas [35]. Reduced red cell indices (MCV and MCH) as observed agrees with findings previously reported [36]. Red cell haemolysis by the spleen and the corresponding urinary iron loss could have accounted for the reduced HGB and RBC indices among the positive cases [34]. Microcythaemia and hypochromia due to reduced MCV and MCHC in *Schistosoma* infected individuals advance the effect of schistosomiasis through its pathogenesis [37]. The aetiology of anaemia in *Schistosoma*-infected populations may be complicated, and it is a substantial contributor to schistosomiasis-specific impairment as it has the ability to impart quality of life [38]. Anaemia has been associated with hereditary haemoglobinopathies, haemorrhage, bacteraemia, micronutrient (such as iron, copper, folate, vitamins A and B12) deficiencies, and parasitic co-infections such as malaria and hookworm [38,39]. A significant rise in the WBC, neutrophiles and monocytes is an indication of a corresponding rise in infection and may lead to a general immunological response as a number of systems employing antibodies are released to disturb the schistosomes [34].

Urine analysis revealed high pus cells and red blood cells counts among *Schistosoma* positive participants when compared to participants without *Schistosoma* Infection. In addition, *Schistosoma* positive participants had significant number being positive for urine protein, blood and leucocytes after biochemical testing using urine dipsticks. Similar to the present finding, increased pus cells were seen in urine samples of 57.5% of *Schistosoma* positive subjects and was linked to the presence of bacterial infection and inflammatory lesion on the bladder caused by *S*. *haematobium* [40]. Haematuria is considered one of the first sign for *Schistosoma* investigation. Haematuria and proteinuria has long been reported as important predictors of *Schistosoma* infection [41]. Pyuria, haematuria, and proteinuria observed in this study are linked to urinary tract infection and renal impairment [42,43]. These findings reveal that *Schistosoma* infection may have resulted in comorbidities and requires immediate clinical attention to be curtailed in the area.

The present study presents important findings necessary for policy formulation and consequent eradication of *Schistosoma* infection at KAPND and other places. However, the study is not without limitations. First, prevalence of *S*. *hematobium* was based on examination of samples in a single day, although earlier report suggests that *S*. *haematobium* egg released may vary per day [44]. This means that the implication of haematological results could be underestimated based on one urine test result. Second, by picking families based on a planned walking route during the workday, our sampling technique may have introduced bias. Again, by interpreting questionnaires for some participants, we may have introduced some level of response bias influenced by participants’ understanding of the interpretations. Our limited sample size may explain the lack of significance in some haematological parameters despite the high prevalence reported. Again, the study design employed limited our ability to obtain reasons behind participants’ KAP. Clearly, a prospective follow-up research with a mix-method study design and larger sample size would be more useful in determining the impact of infection and to get an in-depth information on the communities’ perceptions and views of *S*. *haematobium*. Lastly, assessment of clinical outcomes was based on haematological and urine parameters only. Future studies could look at the effect of schistosomiasis on biochemical parameters such as liver function, kidney function and lipid profile.

## 5. Conclusion

We report that *S*. *haematobium* is endemic in the KAPND of Ghana and is associated with a gap in knowledge, attitudes, and practices of inhabitants. This study found that individuals had inadequate knowledge of schistosomiasis and that the attitudes and practices reported favours the disease’s prevalence. Again, *S*. *haematobium* infection results in poor clinical outcomes and comorbidities by creating an imbalance in the haematological and urine profiles. If not properly managed, these have the potency to impart work capacity, immunogenicity, and cognitive development in these populations. Mass drug administration coupled with nutritional intervention, good water supply, vector control and intensive public health education would be necessary in addressing the prevalence and deleterious effects of *S*. *haematobium* infections in the district. A schistosomiasis education campaign should focus on the causes, transmission, treatment, and prevention of the disease as well as, the impact of the infection on the health and nutrition of the inhabitants.

## Supporting information

S1 File Raw data file. (XLSX)
